# Trends in US Health Insurance Coverage During the COVID-19 Pandemic

**DOI:** 10.1001/jamahealthforum.2021.2487

**Published:** 2021-09-03

**Authors:** M. Kate Bundorf, Sumedha Gupta, Christine Kim

**Affiliations:** 1 Sanford School of Public Policy, Duke University, Durham, North Carolina; 2 National Bureau of Economic Research, Cambridge, Massachusetts; 3 Indiana University–Purdue University Indianapolis, Indianapolis; 4 Stanford University, Stanford, California

## Abstract

**Importance:**

While most working-age adults in the US obtain health insurance through an employer, little is known about the implications of the massive pandemic-related job loss in March 2020 and subsequent rebound for rates of employer-sponsored coverage and uninsurance.

**Objective:**

To determine how health insurance coverage changed during the COVID-19 pandemic.

**Design, Setting, and Participants:**

Analysis of trends in insurance coverage based on repeated cross sections of the US Census Bureau’s Household Pulse Survey data, using linear regression to adjust for respondent’s demographic and socioeconomic characteristics and state of residence. More than 1.2 million US adults aged 18 to 64 years were surveyed from April 23 through December 21, 2020.

**Exposures:**

The COVID-19 pandemic, separated into spring and summer and fall and winter time periods during 2020, as well as state Medicaid expansion status.

**Main Outcomes and Measures:**

Regression-based estimates of the weekly percentage-point change in respondents’ health insurance status, including having any health insurance, any employer-sponsored health insurance, or only nonemployer sponsored coverage. Nonemployer-sponsored coverage is categorized into private, Medicaid, and other public in some analyses.

**Results:**

The study population included 1 212 816 US adults (51% female; mean [SD] age, 42 [13] years) across all 50 US states and Washington DC. Among these respondents, rates of employer-sponsored coverage declined by 0.2 percentage points each week during the COVID-19 pandemic. Other types of coverage, particularly from public sources, increased by 0.1 and 0.2 percentage points in the spring and summer and fall and winter periods, respectively. Overall, health insurance coverage of any type declined, particularly during the spring and summer period, during which uninsurance increased by 1.4 percentage points, representing more than 2.7 million newly uninsured people, over a 12-week period.

**Conclusions and Relevance:**

In this cross-sectional study of data from the US Census Bureau’s Household Pulse Survey, results showed that while public programs played an important role in protecting US adults from pandemic-driven declines in employment-sponsored coverage, many people became uninsured during 2020.

## Key Points

**Question:**

How did health insurance coverage change during the COVID-19 pandemic?

**Findings:**

In this cross-sectional survey study of more than 1.2 million US adults, rates of employer-sponsored coverage declined and rates of other types of coverage increased after the pandemic began and throughout 2020. Rates of uninsurance increased, particularly during the spring and summer.

**Meaning:**

While public programs played an important role in protecting US adults from pandemic-driven declines in employer-sponsored coverage, many people became uninsured during the pandemic.

## Teaser

This cross-sectional study seeks to determine how health insurance coverage changed in the US during the COVID-19 pandemic.

## Introduction

The COVID-19 pandemic has placed millions of workers at risk of uninsurance. The pandemic’s onset was accompanied by an unprecedentedly large, swift decline in employment, with the unemployment rate peaking at 14.7% in April 2020 and steadily declining to 6.7% in December 2020. These rates do not account for those who had dropped out of the labor force.^1^ Because employer-sponsored insurance (ESI) is the primary source of coverage for working-age adults,^2^ losing a job not only leads to loss of income, but may also lead to loss of health insurance. The financial risk of COVID-19–related care heightened the potential negative consequences of uninsurance.

The extent of uninsurance during the COVID-19 recession, however, is largely unknown, primarily owing to a lack of comprehensive, real-time data on coverage.^3^ Yet, this recession differed in important ways from earlier economic downturns, including the swiftness of the initial economic decline and the sensitivity of the recovery to both policy and the epidemiology of COVID-19. We addressed this gap by analyzing the 2020 Household Pulse Survey (HPS),^4^ a US Census Bureau experimental data product intended to provide timely information on the pandemic’s effect on US households. The survey, which has been conducted approximately every 1 to 2 weeks since April 2020, allows us to examine trends in coverage during the pandemic and to provide some of the first evidence on coverage from a large, high-frequency survey designed to be nationally representative. While the HPS has limitations, primarily owing to its low response rate and its lag in data collection relative to the large, initial decline in employment, with 40 000 to 130 000 respondents per week it is perhaps the only source of information on trends in health insurance coverage during the pandemic from a large population-based sample.

We documented the extent to which insurance coverage changed between mid-April and December 2020. The present analysis is based on the premise that pandemic-driven job loss may have led to declines in ESI that lagged the initial employment decline and persisted throughout 2020. In addition, the extent to which declining own ESI was accompanied by rising uninsurance depends on both whether newly unemployed workers had coverage prior to the pandemic and whether people accessed coverage alternatives during the pandemic, including enrolling in coverage through a spouse or other family member, retaining group coverage through COBRA (Consolidated Omnibus Budget Reconciliation Act),^5^ purchasing subsidized or unsubsidized private individual coverage, or enrolling in a public program such as Medicaid. Given Medicaid’s safety-net role, we investigated differences between states that did and did not expand Medicaid through the Affordable Care Act (ACA). We also examined differences by prepandemic family income, age, sex, and race and ethnicity based on evidence of the pandemic’s disproportionate labor market effects across groups.^6^

## Methods

We collected data from the US Census Bureau’s 2020 HPS, which was fielded approximately every 1 to 2 weeks beginning April 23, 2020.^7^ The HPS randomly selects participants using the US Census Bureau’s Master Address File. Sampled households are contacted by either email or cell phone and then directed to an online survey.^7^ The survey, which was designed to produce state-level estimates, includes responses from between approximately 40 000 and 130 000 respondents each week. As anticipated by the survey designers, however, response rates are relatively low at 1.3% to 10.3% per week.^7,8,9^ We limited the sample to people aged 18 to 64 years to focus on those reliant on ESI. Herein we present several analyses comparing the HPS with alternative nationally representative surveys (eTable 1 in the Supplement).

Because this study used deidentified secondary data, it was not deemed human subject research and not subject to review by the institutional review board at Duke University. The study provides information on response rates as advised by the American Association for Public Opinion Research (AAPOR) reporting guideline.^10^

### Measures

We defined any insurance as whether the respondent indicated having any source of coverage. We divided this category into 2 mutually exclusive groups: any ESI and, among those without ESI, any other non-ESI coverage. When comparing expansion and nonexpansion states, we delineated non-ESI into other private, Medicaid, and other public coverage. Details on measure construction and comparisons to other surveys are provided in eFigures 1, 2A, and 2B in the Supplement.

### Statistical Analyses

We estimated the weekly percentage-point change in insurance between April 23 and December 21, 2020. For each coverage type, we regressed an indicator on a continuous measure of calendar week, testing for a linear trend in coverage over the survey period. We separately analyzed 2 time periods: April 23 through July 21, 2020 (spring and summer), and August 19 through December 21, 2020 (fall and winter), for 2 reasons. First, the epidemiologic, policy, and economic environments changed dramatically between the periods. Spring and summer were marked by a flatter pandemic curve, gradual reopenings of state economies, and the operation of the federal Paycheck Protection Program.^11,12,13,14^ In contrast, fall and winter saw a resurgence in COVID-19 cases and deaths, the lifting of many state business closures, school reopenings, and termination of the federal Paycheck Protection Program. Second, the survey instrument changed between the 2 periods, with a nearly month-long gap between them, a shift from weekly to biweekly frequency, and, while the health insurance questions were asked in the same way, they appeared later in the questionnaire, resulting in higher nonresponse rates (eTable 2 in the Supplement).

We estimated linear regression models, adjusting for respondents’ sex, age, race and ethnicity, education, household size, and indicators of any children in the household and state of residence to control for changes over time in sample composition that may not be captured in survey weights (eTable 1 in the Supplement). We applied the replicate weights developed by the US Census Bureau, which adjusted for nonresponse and coverage of the demographics of the US population,^7^ adjusted the standard errors for clustering within state, and reported the coefficient and 95% CIs on the week measure.

We also estimated separate models by state Medicaid expansion status and by respondent sex (male and female), age (18-26, 27-40, 41-50, and 51-64 years), race and ethnicity (Hispanic, non-Hispanic Asian, non-Hispanic Black, and non-Hispanic White), and pretax, prepandemic annual household income reported retrospectively by the respondent (<$50 000, $50 000-99 999, and ≥$100 000). We excluded observations with missing values for health insurance and those from respondents in the state of Nebraska because it underwent ACA Medicaid expansion during the study period. We also excluded those with missing values for family income in analyses by family income (eTable 2 in the Supplement).

The Supplement provides several analyses supporting our approach. First, models estimated with weekly fixed effects rather than the linear trend demonstrate that the linear trend is an appropriate approximation for health insurance trends (eFigure 3 in the Supplement). Second, tests of whether the linear trend differed between the 2 time periods guide the interpretation of the findings (eTable 3 in the Supplement). Finally, rather than dropping those with missing data on insurance, we reestimated the models by coding missing coverage as a separate category, providing evidence that the results were not sensitive to this exclusion (eFigure 4 in the Supplement). All analyses were conducted using Stata MP, version 16.1 (StataCorp), and statistical significance was defined as *P* ≤ .05.

## Results

**Figure 1.**
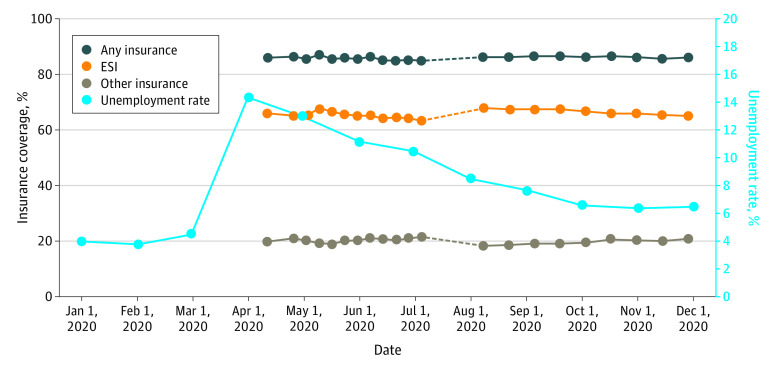
Survey Timing and Trends in Unemployment and Insurance Coverage ESI indicates employer-sponsored insurance.

The study sample, which included 1 212 816 US adults aged 18 to 64 years (51% female; mean [SD] age, 42 [13] years), is similar to the nationally representative American Community Survey and National Health Interview Survey^15^ based on respondent sex, race and ethnicity, and education, but it is slightly older with a larger mean household size (eTable 1 in the Supplement). The HPS was first fielded on April 23, 2020, just after unemployment had peaked (Figure 1). The unemployment rate steadily declined during the remainder of the calendar year. Unadjusted rates of ESI generally declined throughout the year while other coverage generally increased, resulting in either stable or slightly declining rates of any insurance coverage in both time periods. Figure 1 also demonstrates that there were upward and downward shifts in rates of ESI and other coverage, respectively, between the 2 periods. Analyses were conducted separately for each period because it could not be determined whether these shifts were due to survey changes or true coverage changes.

**Figure 2.**
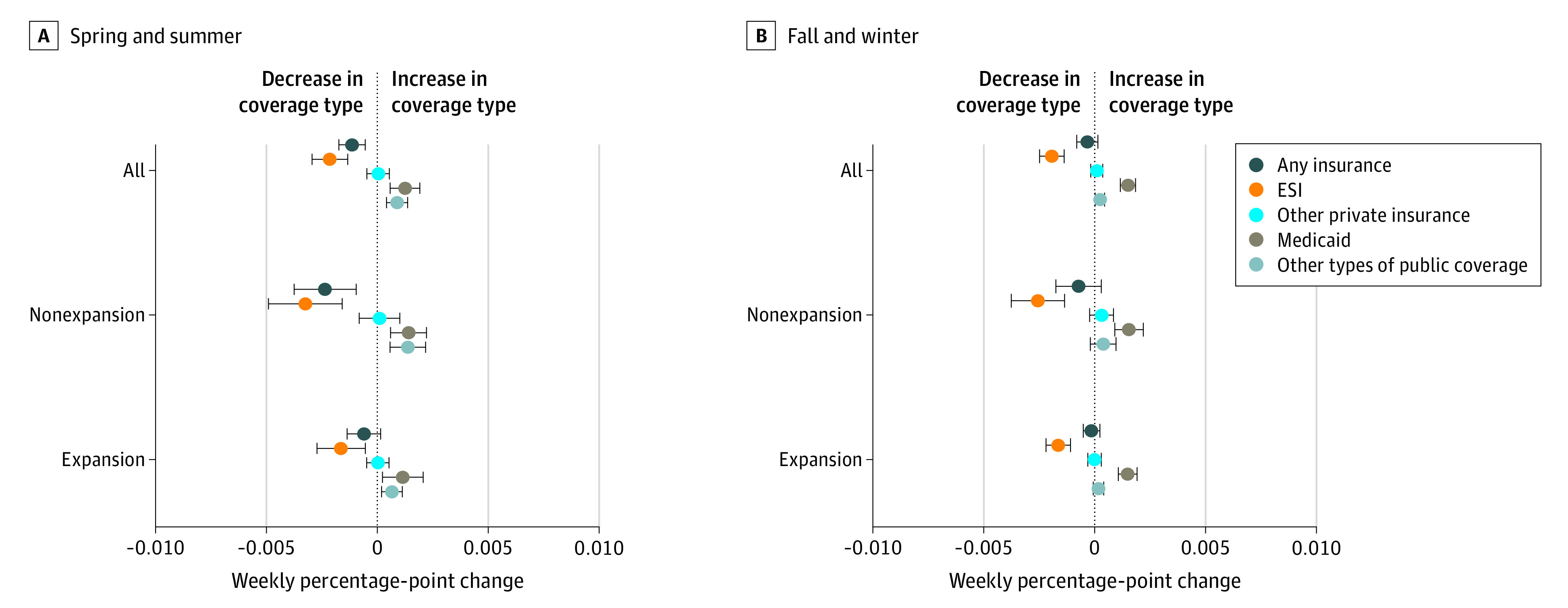
Weekly Percentage-Point Change in 2020 Insurance Coverage, Overall and by State Medicaid Expansion Status Point estimates from regressions are shown, and the error bars indicate 95% CIs. ESI indicates employer-sponsored insurance.

The proportion of people with any type of health insurance decreased by 0.11 percentage points each week during the 12-week period of spring and summer (Figure 2 and eTable 4A in the Supplement), resulting in a 1.36 (0.1137 × 12) percentage-point decline in coverage over the 12-week period. The rise in uninsurance resulted from a 0.21–percentage-point weekly decline in employer-sponsored coverage that was only partly offset by an increase in other coverage.

In contrast, in the fall and winter period the estimate of the decline in any coverage was smaller and not statistically significant; the difference between the 2 periods is statistically significant (eTable 3 in the Supplement). While rates of ESI continued to decline by 0.19 percentage points weekly, the decline was more fully offset by increases in other coverage, primarily Medicaid.

The decline in insurance during spring and summer was concentrated in states not expanding Medicaid. In nonexpansion states, rates of any coverage declined by 0.23 percentage points weekly; rates of ESI declined by 0.32 percentage points per week, while rates of other coverage increased by 0.09 percentage points. In expansion states, in contrast, the overall decline in coverage was small and not statistically significantly different from zero—the combination of a smaller decline in ESI (0.16 percentage points per week) and a similarly sized increase in other coverage as nonexpansion states. In other words, decline in insurance in nonexpansion states was associated with a large decline in ESI, relative to expansion states, that was less fully offset by increases in non-ESI. The increase in non-ESI represented 28% and 63% of the decline in ESI in nonexpansion and expansion states, respectively, during the spring and summer. In both expansion and nonexpansion states, public rather than private coverage was the primary source of coverage gains.

In contrast, during the fall and winter, the differences between expansion and nonexpansion states were less striking (Figure 2). In both types of states, rates of ESI continued to decline but were nearly fully offset by increases in public coverage.

**Figure 3.**
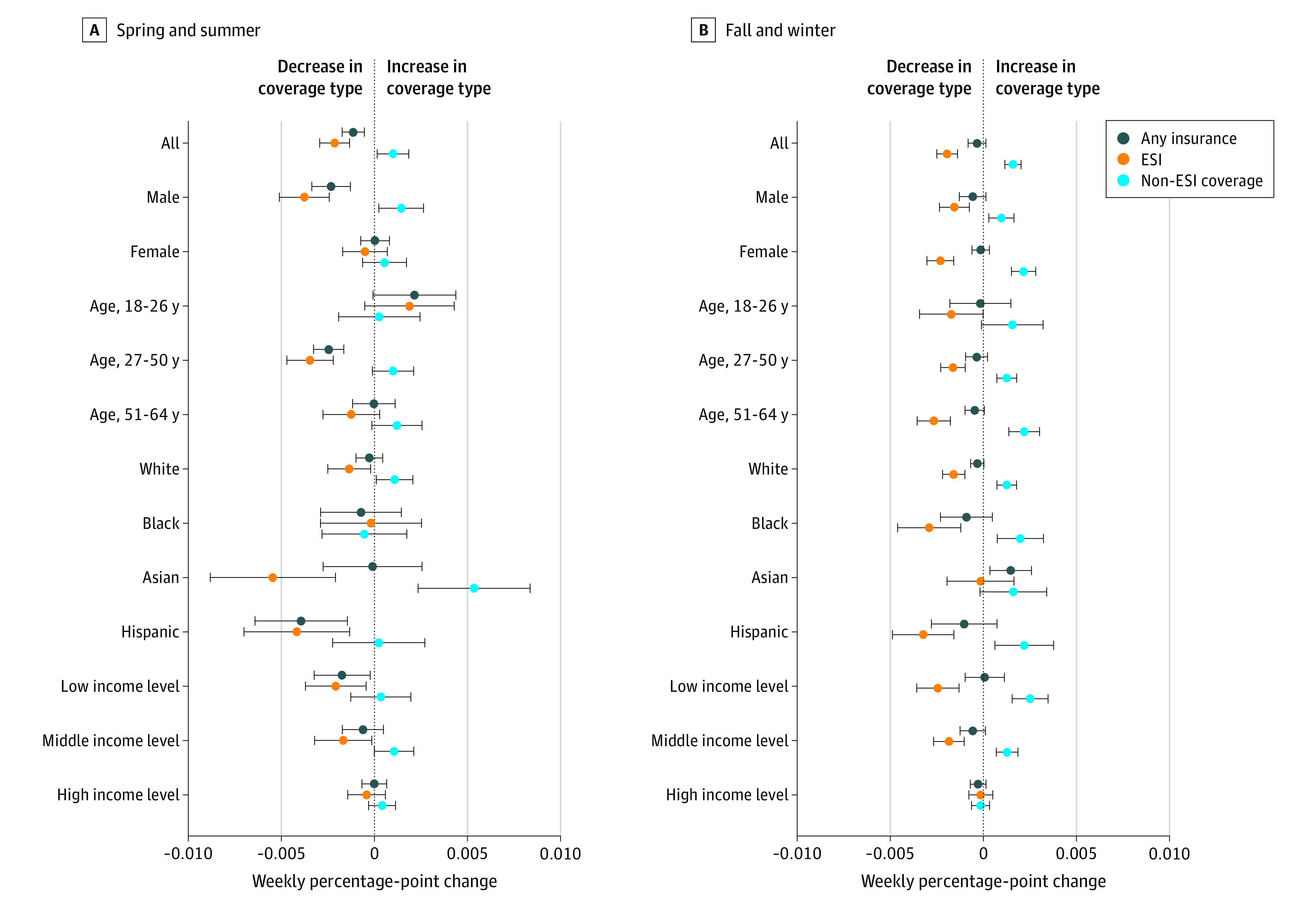
Weekly Percentage-Point Change in 2020 Insurance Coverage, Overall and by Demographic and Socioeconomic Characteristics Point estimates from regressions are shown, and the error bars indicate 95% CIs. ESI indicates employer-sponsored insurance.

The trends varied by population subgroups, particularly in the spring and summer (Figure 3 and eTable 4B in the Supplement). Early in the pandemic, rising uninsurance was concentrated among men, people aged 27 to 50 years, people of Hispanic ethnicity, and people in families with relatively low prepandemic income. For these groups, the decline in ESI was larger than the increase in other sources. People classified in the HPS as Asian and those in the middle family income category also experienced declines in ESI, but they were nearly fully offset by increases in other coverage. In contrast, in the fall and winter declines in rates of ESI were more similar across demographic and socioeconomic subgroups, with nearly every group with the exception of Asian individuals and high prepandemic income households reporting a statistically significant decline (eTable 4B in the Supplement). However, there was not evidence of rising uninsurance in any subgroup due to increases in other sources.

## Discussion

Results of this study provide new evidence on how insurance coverage changed during the COVID-19 pandemic. We found that rates of ESI declined throughout much of 2020, even after the initial shock to employment in March. Because ESI declined throughout the 2 study time periods, a time during which employment was increasing, the results suggest either that ESI declines lagged job loss or that people who returned to work did not necessarily recover their employer-sponsored coverage.

While rates of ESI declined, enrollment in other types of coverage increased. Over the spring and summer period, increases in other types of coverage only partially offset declining ESI, resulting in a 1.36–percentage point increase in uninsurance or approximately 2.7 million newly uninsured people over the 12-week period. The 2.7 million estimate is the product of the number of people 18 to 64 years old in the US in 2019 (202 142 110),^16^ the weekly change in coverage (0.11 percentage points), and the number of weeks (12).

The results indicate that much of the overall decline in coverage took place within a short 3-month period early in the pandemic. While employer-sponsored coverage declined throughout 2020, the decline was more fully offset by increases in other sources later in the year. This could be due to several factors. People may have delayed pursuing alternative sources of coverage early in the pandemic because of substantial uncertainty in the economic environment, such as likely length of unemployment. In addition, it may have taken people time to identify and enroll in alternative coverage sources, particularly because the enrollment process itself may have slowed, given the widespread disruption to businesses and government offices. Finally, the winter surge of COVID-19 cases may have strengthened incentives to obtain coverage as the risk of infection increased. The cross-sectional nature of the HPS does not allow us to determine whether those losing ESI were the same as those gaining other coverage—indeed the gains in non-ESI coverage we observed in the fall and winter period may be responses to coverage loss earlier in the year.

We found that enrollment in public programs, rather than private insurance such as COBRA or on- or off-exchange individual coverage, increased throughout the year. A large increase in Medicaid enrollment was consistent with documentation of rising Medicaid enrollment during the pandemic, as well as prepandemic evidence that the ACA’s new coverage options have alleviated the negative effect of unemployment on insurance coverage.^17,18,19^ The increase in public coverage may also be because of provisions of the Families First Coronavirus Response Act, which prevented states from terminating Medicaid coverage during the pandemic.^20^ In other words, rising rates of Medicaid coverage in both expansion and nonexpansion states may have been driven in part by declining disenrollment due to this policy.

Low take-up of private alternatives may have been driven by their high cost, particularly given the uncertainty over the length of job loss. Many people view extending coverage through COBRA as expensive because most are required to pay the full premium paid by the employer—more than $21 000 annually for family coverage in 2020, plus an administrative fee.^21,22^ People, particularly those without large subsidies, may also view exchange coverage as expensive. Finally, some workers may have been furloughed, retaining coverage until they returned to work. Of course, it is also possible that people either were simply not aware of these options or had private coverage but thought it was public when responding to the survey, particularly in the case of subsidized exchange coverage.

We also documented that, particularly in the early stages, the pandemic had disproportionate associations with coverage across population subgroups. The present results indicate that this issue may be particularly important for Hispanic adults who experienced a decline in ESI without an offsetting increase in non-ESI, resulting in an increase in uninsurance. This raises the concern that the COVID-19 pandemic may have undone some of the reductions in disparities in health insurance generated by the ACA.^23^

The COVID-19 recession, which was initiated by a public health crisis rather than a financial crisis, differs in important ways from the most recent major economic downturn, the Great Recession (2007-2010). In the Great Recession, the association between rising unemployment and uninsurance was concentrated among college-educated, white, older men, and public programs served as a key insurance safety net for women and children.^24^ The COVID-19 recession’s 2-month employment decline, however, was approximately 50% larger than the 2-year employment decline in the Great Recession. In addition, the types of workers losing jobs differed; workers in construction and manufacturing were most affected in the Great Recession, while job loss was concentrated among low-wage service workers in the COVID-19 recession.^25^ Not only did the nature of job loss differ, but newly unemployed workers had more sources of subsidized insurance available to them in the COVID-19 recession. Results of this study suggest that Medicaid, in particular, played an important safety-net role for a broader population, likely because more people are now eligible for Medicaid than in 2007 through 2009. Whether Medicaid coverage continues to expand in 2021 depends on the extent to which employment gains affect Medicaid eligibility. In addition, the American Rescue Plan considerably expanded subsidies for both exchange and COBRA coverage, creating additional alternatives for people without ESI^26^ and suggesting that the private plans may begin to play a more important role.

Looking ahead, unemployment rates are currently much lower than the pandemic’s peak (5.8% in May 2021 vs 14.7% in April 2020) but still above the prepandemic levels, and employment recovery has been lagging among racial and ethnic minority subpopulations.^6,27^ These trends point to the continued importance of safety-net programs both in providing coverage for unemployed workers and in addressing insurance disparities.

### Limitations

This study has several limitations. The HPS’s low response rate, as well as the use of alternative contact modalities including email and texts, raises concern over sample representativeness. The weighted HPS sample is similar to the American Community Survey (eTable 1 in the Supplement) along some dimensions but not others. While rates of insurance coverage differ from those of other surveys, we note that the differences are consistent across states (eFigures 1 and 2 in the Supplement). The present analyses also may miss some of the pandemic’s early effect on coverage given the timing of the HPS’s initial wave relative to the timing of pandemic-related job loss (Figure 1). We also emphasize that the analyses are descriptive. We expect that changes in ESI during the COVID recession were driven by changes in employment. However, the present data do not allow us to link insurance coverage and employment at the individual level. Finally, we note that we interpreted the change in coverage between the spring and summer and fall and winter phases of the survey as driven primarily by the change in survey design and the resulting lower response rate for health insurance questions. It is possible that coverage changed in this short time period in ways that the survey does not allow us to track with the result that the findings may not be representative of the total change in coverage over the study period.

## Conclusions

Overall, results of this cross-sectional study indicate that while public programs played an important role in insulating US adults from pandemic-driven declines in ESI, many became uninsured. Monitoring and strengthening the health insurance safety net will continue to be a policy challenge during the COVID-19 pandemic and beyond.

## Notes

Supplement. eTable 1. Comparison of the Weighted Estimates of Demographic Characteristics of Respondents in the 2018 and 2019 ACS, 2018 NHIIS, and the 2020 HPS eFigure 1. Comparison of Distribution of Health Insurance Coverage between the 2019 ACS and the 2020 HPS eFigure 2A. Comparison of Distribution of Health Insurance Coverage between the 2019 ACS and HPS Spring/Summer 2020 Period by State eFigure 2B. Comparison of Distribution of Health Insurance Coverage between the 2019 ACS and HPS Fall/Winter 2020 Period by State eTable 2. Rates of Missing Data from the Household Pulse Survey for Key Study Variables by Week eFigure 3. Estimates of Insurance Coverage in the HPS by Week and Type of Insurance eTable 3. Test of Difference in Time Trend of Health Insurance Coverage by Survey Period eFigure 4A. Sensitivity of Results to Dropping Missing Data, Expansion and Non-Expansion States eFigure 4B. Sensitivity of Results to Dropping Missing Data, Demographic and Socioeconomic Characteristics eTable 4A. Underlying Data for Estimates Presented in Figure 2 eTable 4B. Underlying Data for Estimates Presented in Figure 3 eReferences
