# Supplementary material for: Trends in US Health Insurance Coverage During the COVID-19 Pandemic
Source: JAMA Health Forum. 2021 Sep 3;2(9):e212487. doi: 10.1001/jamahealthforum.2021.2487 (PMC8796896; doi:10.1001/jamahealthforum.2021.2487)
Supplement: Supplement. — eTable 1. Comparison of the Weighted Estimates of Demographic Characteristics of Respondents in the 2018 and 2019 ACS, 2018 NHIIS, and the 2020 HPS eFigure 1. Comparison of Distribution of Health Insurance Coverage between the 2019 ACS and the 2020 HPS eFigure 2A. Comparison of Distribution of Health Insurance Coverage between the 2019 ACS and HPS Spring/Summer 2020 Period by State eFigure 2B. Comparison of Distribution of Health Insurance Coverage between the 2019 ACS and HPS Fall/Winter 2020 Period by State eTable 2. Rates of Missing Data from the Household Pulse Survey for Key Study Variables by Week eFigure 3. Estimates of Insurance Coverage in the HPS by Week and Type of Insurance eTable 3. Test of Difference in Time Trend of Health Insurance Coverage by Survey Period eFigure 4A. Sensitivity of Results to Dropping Missing Data, Expansion and Non-Expansion States eFigure 4B. Sensitivity of Results to Dropping Missing Data, Demographic and Socioeconomic Characteristics eTable 4A. Underlying Data for Estimates Presented in Figure 2 eTable 4B. Underlying Data for Estimates Presented in Figure 3 eReferences [file jamahealthforum-e212487-s001.pdf]

Supplemental Online Content

Bundorf MK, Gupta S, Kim C. Trends in US health insurance coverage during the COVID-19 pandemic. *JAMA Health Forum*. 2021;2(9):e212487. doi:10.1001/jamahealthforum.2021.2487

eTable 1. Comparison of the Weighted Estimates of Demographic Characteristics of Respondents in the 2018 and 2019 ACS, 2018 NHIIS, and the 2020 HPS

eFigure 1. Comparison of Distribution of Health Insurance Coverage between the 2019 ACS and the 2020 HPS

eFigure 2A. Comparison of Distribution of Health Insurance Coverage between the 2019 ACS and HPS Spring/Summer 2020 Period by State

eFigure 2B. Comparison of Distribution of Health Insurance Coverage between the 2019 ACS and HPS Fall/Winter 2020 Period by State

eTable 2. Rates of Missing Data from the Household Pulse Survey for Key Study Variables by Week

eFigure 3. Estimates of Insurance Coverage in the HPS by Week and Type of Insurance

eTable 3. Test of Difference in Time Trend of Health Insurance Coverage by Survey Period

eFigure 4A. Sensitivity of Results to Dropping Missing Data, Expansion and Non-Expansion States

eFigure 4B. Sensitivity of Results to Dropping Missing Data, Demographic and Socioeconomic Characteristics

eTable 4A. Underlying Data for Estimates Presented in Figure 2

eTable 4B. Underlying Data for Estimates Presented in Figure 3

eReferences

This supplemental material has been provided by the authors to give readers additional information about their work.

© 2021 Bundorf MK et al. *JAMA Health Forum*.

Representativeness of the HPS Sample

The HPS' low response rate, as well as the use of alternative contact modalities including e-mail and texts, raises concern over the non-representativeness of the sample. While the U.S. Census designed the survey with the objective of obtaining nationally representative estimates, we note that, even after applying replicate weights, the HPS sample differs from that of the 2019 ACS. Younger people, those with higher incomes and those in smaller households are underrepresented in the HPS, even after applying the weights (see Appendix Table 1). We note that information on income is elicited differently in the HPS than in other surveys. In each HPS wave, respondents are asked about their pre-pandemic income, resulting in an increasingly long retrospection period as the survey progresses. In addition, HPS respondents have higher rates of both employer-sponsored and other types of public and private coverage and are more likely to be uninsured than those in the 2019 ACS (See Appendix eFigure 1) and these differences are largely consistent across states (See Appendix eFigure 2). While these differences in insurance coverage could be caused by the pandemic due to the timing of the two surveys, they are more likely to be driven by selection into the HPS. We address this by focusing on changes over time within the HPS sample in our analysis.

© 2021 Bundorf MK et al. *JAMA Health Forum*.

eTable 1: Comparison of the Weighted Estimates of Demographic Characteristics of Respondents in the 2018 and 2019 ACS, 2018 NHIIS, and the 2020 HPS

	ACS		NHIS	HPS			
				Spring/Summer 2020		Fall/Winter 2020	
	2018	2019	2018	All	Excl. missing HI	All	Excl. missing HI
Female	0.5022	0.5021	0.5082	0.5083	0.5090	0.5083	0.5141
	(0.0005)	(0.0005)	(0.0026)	(0.0002)	(0.0005)	(0.0002)	(0.0008)
Age 18-26	0.1985	0.1965	0.1947	0.1433	0.1278	0.1574	0.1316
	(0.0004)	(0.0004)	(0.0022)	(0.0009)	(0.0010)	(0.0008)	(0.0009)
Age 27-40	0.3077	0.3113	0.3059	0.3436	0.3462	0.3282	0.3288
	(0.0004)	(0.0005)	(0.0024)	(0.0009)	(0.0011)	(0.0011)	(0.0012)
Age 41-50	0.2013	0.2011	0.2037	0.2101	0.2156	0.2102	0.2184
	(0.0004)	(0.0004)	(0.0021)	(0.0007)	(0.0009)	(0.0008)	(0.0009)
Age 51-64	0.2924	0.2911	0.2957	0.3030	0.3104	0.3041	0.3212
	(0.0004)	(0.0004)	(0.0023)	(0.0006)	(0.0007)	(0.0005)	(0.0008)
Non-Hispanic White	0.5964	0.5921	0.5993	0.5862	0.5957	0.5867	0.6073
	(0.0005)	(0.0005)	(0.0026)	(0.0003)	(0.0007)	(0.0003)	(0.0009)
Non-Hispanic Black	0.1281	0.1287	0.1248	0.1268	0.1230	0.1238	0.1153
	(0.0004)	(0.0004)	(0.0018)	(0.0004)	(0.0006)	(0.0004)	(0.0006)
Non-Hispanic Asian	0.0632	0.0636	0.0643	0.0558	0.0555	0.0565	0.0562
	(0.0002)	(0.0002)	(0.0013)	(0.0006)	(0.0006)	(0.0004)	(0.0005)
Non-Hispanic Other	0.0300	0.0306	0.0276	0.0419	0.0417	0.0414	0.0415
	(0.0002)	(0.0002)	(0.0009)	(0.0005)	(0.0005)	(0.0005)	(0.0005)
Hispanic	0.1823	0.1850	0.1839	0.1893	0.1841	0.1916	0.1797
	(0.0004)	(0.0004)	(0.0020)	(0.0003)	(0.0005)	(0.0003)	(0.0008)
High School Diploma	0.6034	0.5968	0.5501	0.6007	0.5876	0.5972	0.5678
	(0.0005)	(0.0005)	(0.0026)	(0.0008)	(0.0011)	(0.0006)	(0.0009)
College Degree	0.2869	0.2922	0.3343	0.2700	0.2766	0.2765	0.2924
	(0.0004)	(0.0004)	(0.0025)	(0.0012)	(0.0013)	(0.0007)	(0.0009)
Graduate Degree	0.1097	0.1110	0.1156	0.1292	0.1359	0.1264	0.1398
	(0.0003)	(0.0003)	(0.0017)	(0.0006)	(0.0007)	(0.0004)	(0.0005)
Low income	0.3455	0.3248	0.3044	0.1684	0.1682	0.1436	0.1433
	(0.0005)	(0.0005)	(0.0024)	(0.0014)	(0.0014)	(0.0011)	(0.0012)
Middle income	0.2788	0.2769	0.2747	0.2383	0.2381	0.2240	0.2240
	(0.0004)	(0.0004)	(0.0023)	(0.0014)	(0.0014)	(0.0013)	(0.0013)
High income	0.3643	0.3869	0.3196	0.1722	0.1723	0.1710	0.1708
	(0.0004)	(0.0005)	(0.0024)	(0.0012)	(0.0012)	(0.0009)	(0.0009)
Household Size	2.9769	2.9762	3.1455	3.6285	3.5928	3.5806	3.5154
	(0.0016)	(0.0016)	(0.0084)	(0.0147)	(0.0152)	(0.0134)	(0.0122)
Children in household	0.4057	0.4030	0.4476	0.4582	0.4535	0.4600	0.4512
	(0.0005)	(0.0005)	(0.0026)	(0.0016)	(0.0016)	(0.0011)	(0.0015)
Missing Income				0.1398	0.0388	0.2980	0.0972
				(0.0018)	(0.0007)	(0.0015)	(0.0010)
Missing Health Ins.				0.1063	0.0000	0.2241	0.0000
				(0.0015)		(0.0013)	
N	1924001	1921823	42994	820833	740819	589129	491560

© 2021 Bundorf MK et al. *JAMA Health Forum*.

Note: State identifiers are not available for the NHIS data from IPUMS. To facilitate comparison, unlike our main analysis with the HPS sample, observations for Nebraska are retained in all 3 samples – ACS, NHIS and the HPS – in this table. The HPS sample with Nebraska includes 1232379 observations. The HPS sample without Nebraska, used for our main analysis, includes N= 1212816.

© 2021 Bundorf MK et al. *JAMA Health Forum*.

eFigure 1: Comparison of Distribution of Health Insurance Coverage between the 2019 ACS and the 2020 HPS

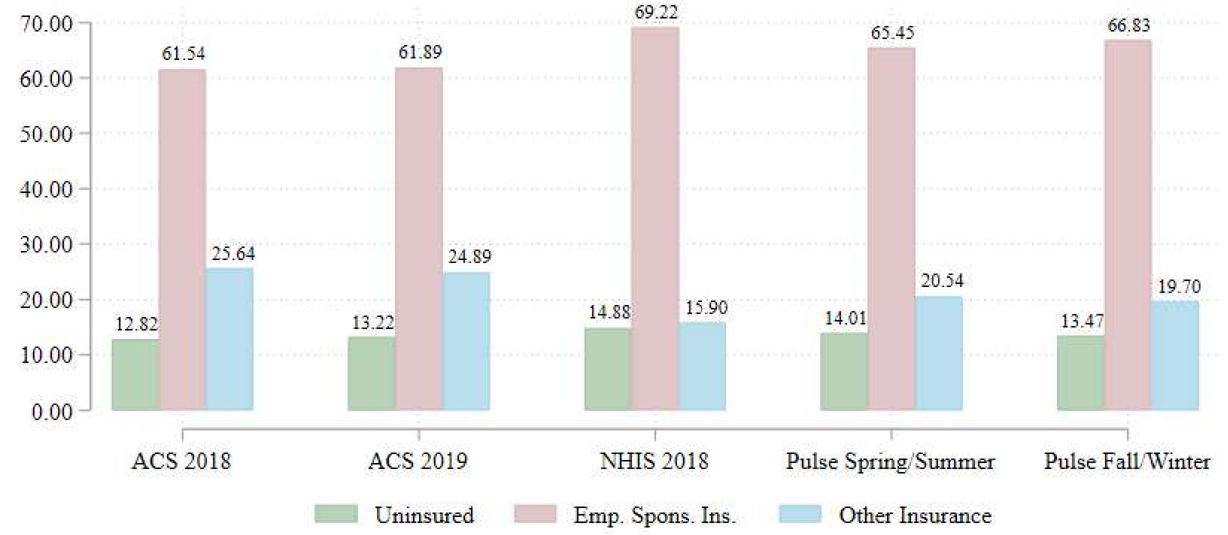

Note: Author's calculations of health insurance coverage in American Community Survey, 2018 and 2019, National Health Interview Survey, 2018, and U.S. Census Household Pulse Survey (April 23-December 21, 2020). The uninsured constituted 12.82 percent [95% CI, 12.76 - 12.89] in ACS 2018, 13.22 percent [95% CI, 13.15 - 13.29] in ACS 2019, and 14.88 percent [95% CI, 14.51 - 15.25] in NHIS 2018, compared with 14.01 percent [95% CI, 13.75 - 14.27] in the Pulse during spring/summer and 13.47 [95% CI, 13.22 - 13.72] during fall/winter of 2020. Employer sponsored insurance constituted 61.54 percent [95% CI, 61.45 - 61.63] in ACS 2018, 61.89 percent [95% CI, 61.80 - 61.99] in ACS 2019 and 69.22 percent [95% CI, 68.74 - 69.69] in NHIS 2018, compared with 65.45 percent [95% CI, 65.12 - 65.78] in Pulse during spring/summer and 66.83 percent [95% CI, 66.53 - 67.14] during fall/winter of 2020. Other insurance constituted 25.64 percent [95% CI, 25.55 - 25.72] in ACS2018, 24.89 percent [95% CI, 24.80 - 24.97] in ACS2019 and 15.90 percent [95% CI, 15.52 - 16.28] in NHIS 2018, compared with 20.54 percent [95% CI, 20.27 - 20.82] in Pulse during spring/summer and 19.70 percent [95% CI, 19.44 - 19.96] during fall/winter of 2020.
Source: ACS 2018 and 2019, NHIS 2018, and U.S. Census Household Pulse Survey (April 23-December 21, 2020).

© 2021 Bundorf MK et al. *JAMA Health Forum*.

eFigure 2A: Comparison of Distribution of Health Insurance Coverage between the 2019 ACS and HPS Spring/Summer 2020 Period by State

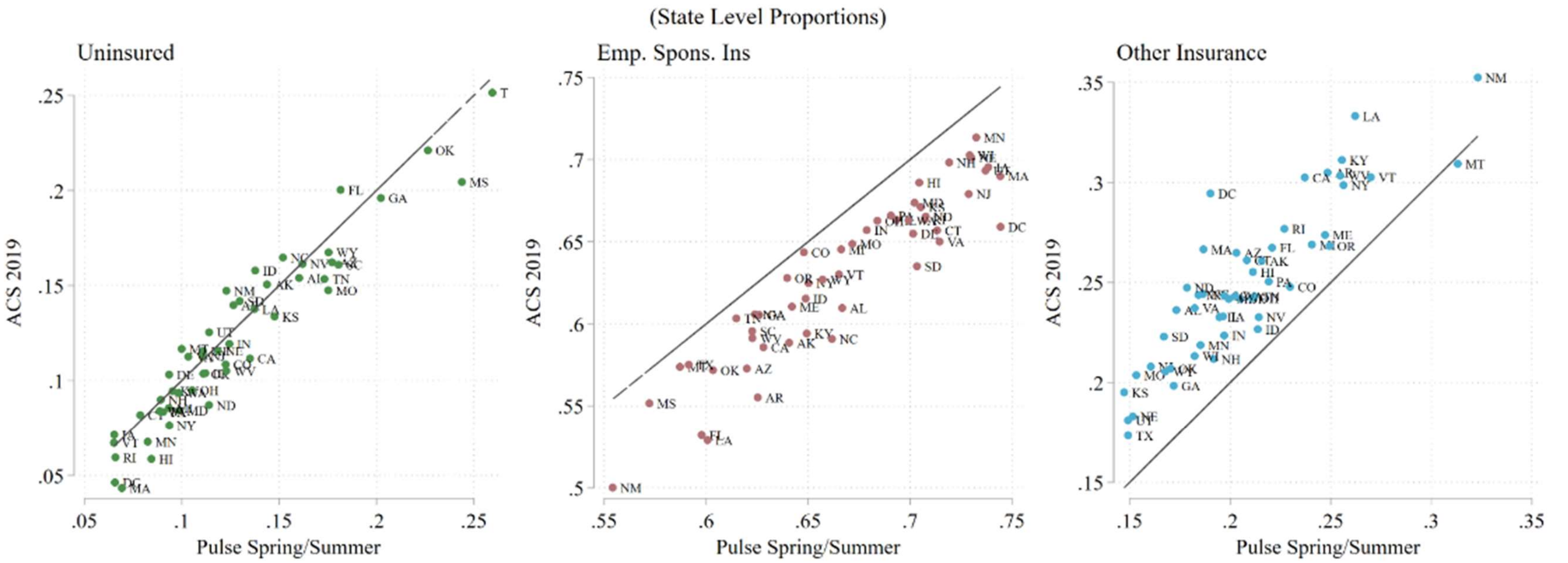

Note: Author's calculations of health insurance coverage in the fifty U.S. states in the American Community Survey, 2019 and the U.S. Census Household Pulse Survey (April 23-July 21, 2020). Forty-five degree line presented for comparison.
Source: ACS 2019 and U.S. Census Household Pulse Survey (April 23-July 21, 2020).

© 2021 Bundorf MK et al. *JAMA Health Forum*.

eFigure 2B: Comparison of Distribution of Health Insurance Coverage between the 2019 ACS and HPS Fall/Winter 2020 Period by State

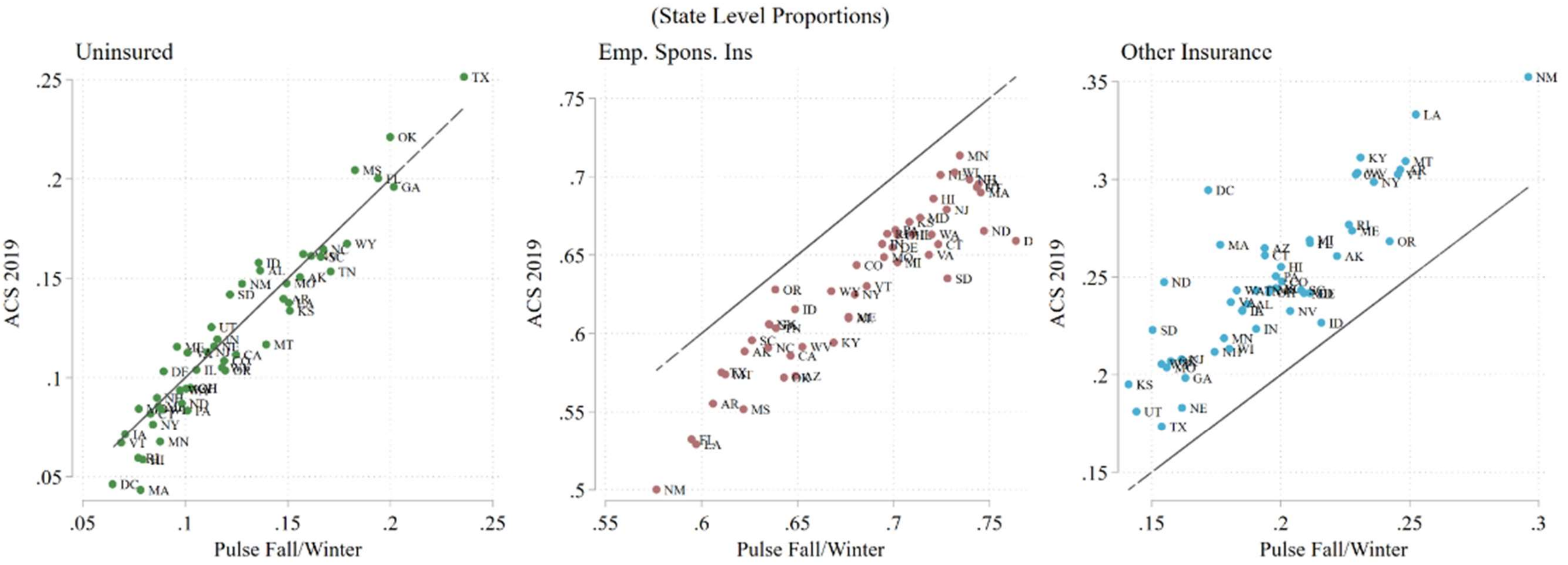

Note: Author's calculations of health insurance coverage in the fifty U.S. states in the American Community Survey, 2019 and the U.S. Census Household Pulse Survey (August 19-December 21, 2020). Forty-five degree line presented for comparison.
Source: ACS 2019 and U.S. Census Household Pulse Survey (August 19-December 21, 2020).

© 2021 Bundorf MK et al. *JAMA Health Forum*.

Measurement of Health Insurance Coverage

Following the design of insurance coverage questions in the National Health Interview Survey,[1] the HPS survey instrument asks respondents, "are you currently covered by any of the following types of health insurance or health coverage plans?" The potential responses are 1) Insurance through a current or former employer or union of yours or another family member, 2) Insurance purchased directly from an insurance company by you or another family member, 3) TRICARE or other military health care, 4) Medicaid, Medical Assistance, or any kind of government-assistance plan for those with low incomes or a disability, 5) Medicare, for people 65 and older, or people with certain disabilities, 6) The Veteran's Administration, meaning you are currently enrolled for VA health care, 7) Indian Health Service, or 8) Other health insurance or health coverage plan. The potential responses are yes, no, or don't know.

We develop a measure of "any insurance" based on whether the respondent indicated that they had coverage types 1) to 6). We then divide this category into two mutually exclusive groups, distinguishing between any employer-sponsored coverage (ESI) (response 1) and other type of coverage (responses 2-6) but no ESI (non-ESI). In some analyses, we delineate "non-ESI" into other private (response 2), Medicaid (response 4), and other publicly funded (responses 3, 5 or 6).

We define health insurance coverage as missing if a respondent did not respond either yes or no to any category. Consistent with coding from the NHIS, we do not code those indicating category 7 or 8 as having coverage,[2] although this decision does not have a substantive effect on our findings.

© 2021 Bundorf MK et al. *JAMA Health Forum*.

eTable 2: Rates of Missing Data from the Household Pulse Survey for Key Study Variables by Week

VARIABLES	Low Income	Middle Income	High Income	Income Missing	ESI	Non-ESI	Uninsured	Insurance Missing	Married	Not Married	Marital Status Missing
Apr 23-May 5	-0.018***	0.014***	0.011**	-0.007	0.023***	-0.014**	-0.011**	0.003	0.017***	-0.018***	0.002*
	(0.006)	(0.005)	(0.004)	(0.004)	(0.005)	(0.005)	(0.005)	(0.003)	(0.005)	(0.005)	(0.001)
May 7-May 12	0.003	0.019***	0.018***	-0.040***	0.028***	-0.000	-0.011**	-0.017***	0.028***	-0.027***	-0.001
	(0.007)	(0.007)	(0.004)	(0.005)	(0.008)	(0.006)	(0.005)	(0.005)	(0.007)	(0.007)	(0.001)
May 14-May 19	-0.014**	0.005	0.014***	-0.005	0.012*	-0.013**	-0.007	0.008*	-0.013**	0.013**	-0.000
	(0.006)	(0.006)	(0.004)	(0.005)	(0.007)	(0.005)	(0.005)	(0.004)	(0.005)	(0.005)	(0.001)
May 21-May 26	-0.047***	0.005	0.020***	0.022***	0.019***	-0.025***	-0.025***	0.031***	0.004	-0.005	0.001
	(0.006)	(0.006)	(0.005)	(0.004)	(0.006)	(0.005)	(0.005)	(0.004)	(0.006)	(0.005)	(0.001)
May 28-Jun 2	-0.042***	0.003	0.014***	0.026***	0.008	-0.027***	-0.012***	0.031***	-0.003	0.002	0.000
	(0.006)	(0.005)	(0.004)	(0.004)	(0.006)	(0.005)	(0.004)	(0.003)	(0.005)	(0.005)	(0.001)
Jun 4-Jun 9	-0.038***	0.004	0.009**	0.025***	0.002	-0.017***	-0.013***	0.028***	-0.011*	0.010*	0.001
	(0.005)	(0.005)	(0.004)	(0.005)	(0.005)	(0.006)	(0.005)	(0.003)	(0.006)	(0.006)	(0.001)
Jun 11-Jun 16	-0.013**	0.009	0.008**	-0.004	0.022***	-0.008	-0.005	-0.009**	0.011**	-0.010*	-0.001
	(0.006)	(0.006)	(0.004)	(0.004)	(0.006)	(0.006)	(0.006)	(0.004)	(0.005)	(0.005)	(0.001)
Jun 18-Jun 23	-0.011*	0.012**	0.010*	-0.011**	0.020***	-0.003	-0.011**	-0.006*	0.003	-0.001	-0.002**
	(0.006)	(0.005)	(0.005)	(0.004)	(0.006)	(0.005)	(0.005)	(0.003)	(0.005)	(0.005)	(0.001)
Jun 25-Jun 30	0.000	0.006	0.006	-0.012***	0.012**	-0.004	-0.001	-0.007*	0.003	-0.003	-0.000
	(0.006)	(0.006)	(0.004)	(0.004)	(0.005)	(0.004)	(0.005)	(0.003)	(0.006)	(0.006)	(0.001)
Jul 2-Jul 7	-0.006	0.002	0.009**	-0.005	0.012**	-0.009**	-0.001	-0.001	0.005	-0.005	0.000
	(0.006)	(0.006)	(0.004)	(0.005)	(0.006)	(0.004)	(0.006)	(0.004)	(0.006)	(0.005)	(0.001)
Jul 9-Jul 14	-0.010*	0.009*	0.006	-0.005	0.006	-0.004	-0.004	0.002	0.008	-0.008	-0.000
	(0.005)	(0.005)	(0.004)	(0.004)	(0.005)	(0.005)	(0.004)	(0.003)	(0.005)	(0.005)	(0.001)
Aug 19-Aug 31	-0.110***	-0.031***	-0.016***	0.156***	-0.039***	-0.050***	-0.028***	0.118***	0.007	-0.009**	0.002***
	(0.005)	(0.005)	(0.004)	(0.005)	(0.006)	(0.005)	(0.004)	(0.004)	(0.005)	(0.004)	(0.001)
Sep 2-Sep 14	-0.105***	-0.032***	-0.009**	0.146***	-0.039***	-0.046***	-0.027***	0.113***	0.005	-0.010**	0.005***
	(0.005)	(0.005)	(0.004)	(0.005)	(0.006)	(0.005)	(0.004)	(0.004)	(0.004)	(0.004)	(0.001)
Sep 16-Sep 28	-0.100***	-0.033***	-0.009**	0.143***	-0.037***	-0.042***	-0.030***	0.110***	0.003	-0.005	0.002**
	(0.006)	(0.005)	(0.004)	(0.005)	(0.006)	(0.004)	(0.004)	(0.004)	(0.004)	(0.004)	(0.001)
Sep 30-Oct 12	-0.108***	-0.031***	-0.011***	0.150***	-0.042***	-0.044***	-0.030***	0.116***	0.008	-0.009*	0.001*
	(0.006)	(0.005)	(0.004)	(0.006)	(0.006)	(0.006)	(0.004)	(0.005)	(0.005)	(0.005)	(0.001)

© 2021 Bundorf MK et al. *JAMA Health Forum*.

VARIABLES	Low Income	Middle Income	High Income	Income Missing	ESI	Non-ESI	Uninsured	Insurance Missing	Married	Not Married	Marital Status Missing
Oct 14-Oct 26	-0.106***	-0.038***	-0.008**	0.152***	-0.051***	-0.042***	-0.028***	0.122***	0.014***	-0.018***	0.004***
	(0.006)	(0.005)	(0.004)	(0.006)	(0.005)	(0.005)	(0.004)	(0.005)	(0.005)	(0.005)	(0.001)
Oct 28-Nov 9	-0.121***	-0.040***	-0.012**	0.173***	-0.070***	-0.037***	-0.034***	0.141***	0.029***	-0.030***	0.001
	(0.006)	(0.006)	(0.005)	(0.007)	(0.006)	(0.005)	(0.006)	(0.006)	(0.006)	(0.006)	(0.001)
Nov 11-Nov 23	-0.117***	-0.035***	-0.020***	0.172***	-0.063***	-0.037***	-0.030***	0.131***	0.015***	-0.018***	0.003***
	(0.005)	(0.006)	(0.004)	(0.006)	(0.006)	(0.006)	(0.005)	(0.005)	(0.005)	(0.006)	(0.001)
Nov 25-Dec 7	-0.106***	-0.043***	-0.011**	0.160***	-0.065***	-0.038***	-0.026***	0.129***	0.020***	-0.022***	0.003***
	(0.006)	(0.005)	(0.004)	(0.006)	(0.006)	(0.005)	(0.005)	(0.005)	(0.005)	(0.005)	(0.001)
Dec 9-Dec 21	-0.100***	-0.043***	-0.017***	0.160***	-0.068***	-0.032***	-0.028***	0.128***	0.011**	-0.016***	0.005***
	(0.006)	(0.005)	(0.004)	(0.006)	(0.006)	(0.005)	(0.005)	(0.005)	(0.005)	(0.005)	(0.001)
Constant	0.366***	0.252***	0.241***	0.141***	0.571***	0.194***	0.134***	0.101***	0.514***	0.480***	0.006***
	(0.005)	(0.004)	(0.003)	(0.004)	(0.004)	(0.004)	(0.004)	(0.003)	(0.004)	(0.003)	(0.001)
Observations	1,409,962	1,409,962	1,409,962	1,409,962	1,409,962	1,409,962	1,409,962	1,409,962	1,409,962	1,409,962	1,409,962
R-squared	0.010	0.003	0.001	0.039	0.005	0.002	0.001	0.027	0.000	0.000	0.000
F-statistic	176.7	34.37	14.92	292.7	56.64	18.19	15.79	265.3	7.431	8.685	9.781
p-value	0	0	0	0	0	0	0	0	6.57e-10	0	0

Standard errors in parentheses
*** p<0.01, ** p<0.05, * p<0.1

© 2021 Bundorf MK et al. *JAMA Health Forum*.

eFigure 3: Estimates of Insurance Coverage in the HPS by Week and Type of Insurance

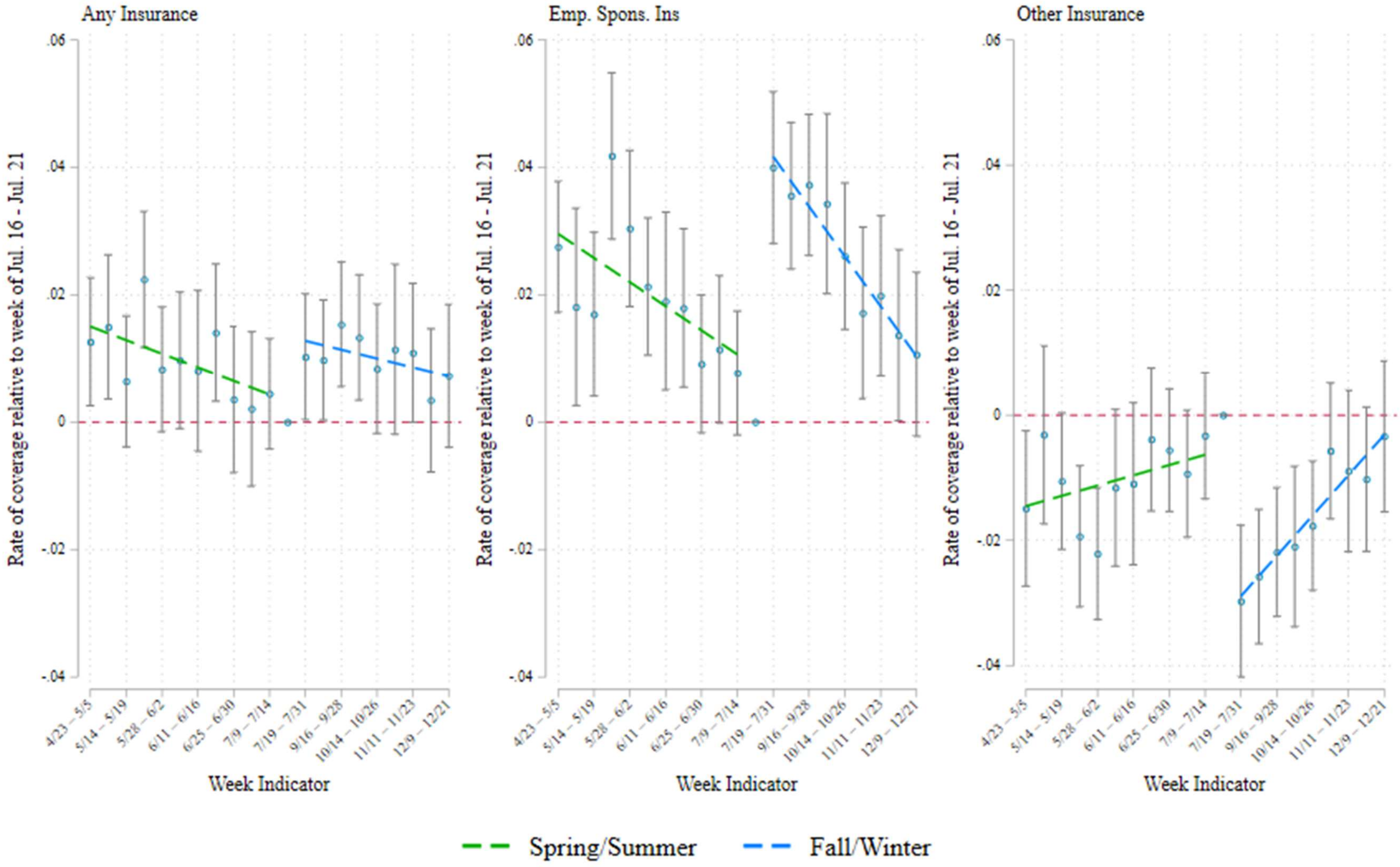

Note: Author's calculations of week fixed effects health insurance coverage in the fifty U.S. states in the U.S. Census Household Pulse Survey (April 23-December 21, 2020).
Source: U.S. Census Household Pulse Survey (April 23-December 21, 2020).

© 2021 Bundorf MK et al. *JAMA Health Forum*.

eTable 3: Test of Difference in Time Trend of Health Insurance Coverage by Survey Period

	(1)<br>Any<br>Insurance	(2)<br>ESI	(3)<br>Non-ESI
All			
Week	-0.00113***	-0.00210***	0.000973**
	(0.00030)	(0.00040)	(0.00042)
Fall/Winter Period	0.00264	0.0432***	-0.0406***
	(0.00631)	(0.00772)	(0.00666)
Week * Fall/Winter Period	0.000777*	0.000141	0.000636
	(0.00040)	(0.00049)	(0.00051)
Observations	1212816	1212816	1212816
Non-Expansion States			
Week	-0.00232***	-0.00320***	0.000882
	(0.00069)	(0.00083)	(0.00063)
Fall/Winter Period	0.00426	0.0448***	-0.0405***
	(0.01396)	(0.01530)	(0.01105)
Week * Fall/Winter Period	0.00159*	0.000618	0.000971
	(0.00095)	(0.00104)	(0.00078)
Observations	296420	296420	296420
Expansion States			
Week	-0.000602	-0.00160***	0.000994*
	(0.00038)	(0.00054)	(0.00054)
Fall/Winter Period	0.00141	0.0424***	-0.0410***
	(0.00565)	(0.00875)	(0.00847)
Week * Fall/Winter Period	0.000439	-0.0000762	0.000515
	(0.00043)	(0.00062)	(0.00063)
Observations	916396	916396	916396

Note: Table presents the results of a regression of coverage type on calendar week, an indicator of survey period and the interaction of the two variables as well as all the control variables included in the main models. The data are pooled across the two survey periods. Standard errors are in parentheses and are clustered by state. Estimates are weighted to be nationally representative using HPS replicate weights. *** p<0.01, ** p<0.05, * p<0.1

© 2021 Bundorf MK et al. *JAMA Health Forum*.

eFigure 4A: Sensitivity of Results to Dropping Missing Data, Expansion and Non-Expansion States

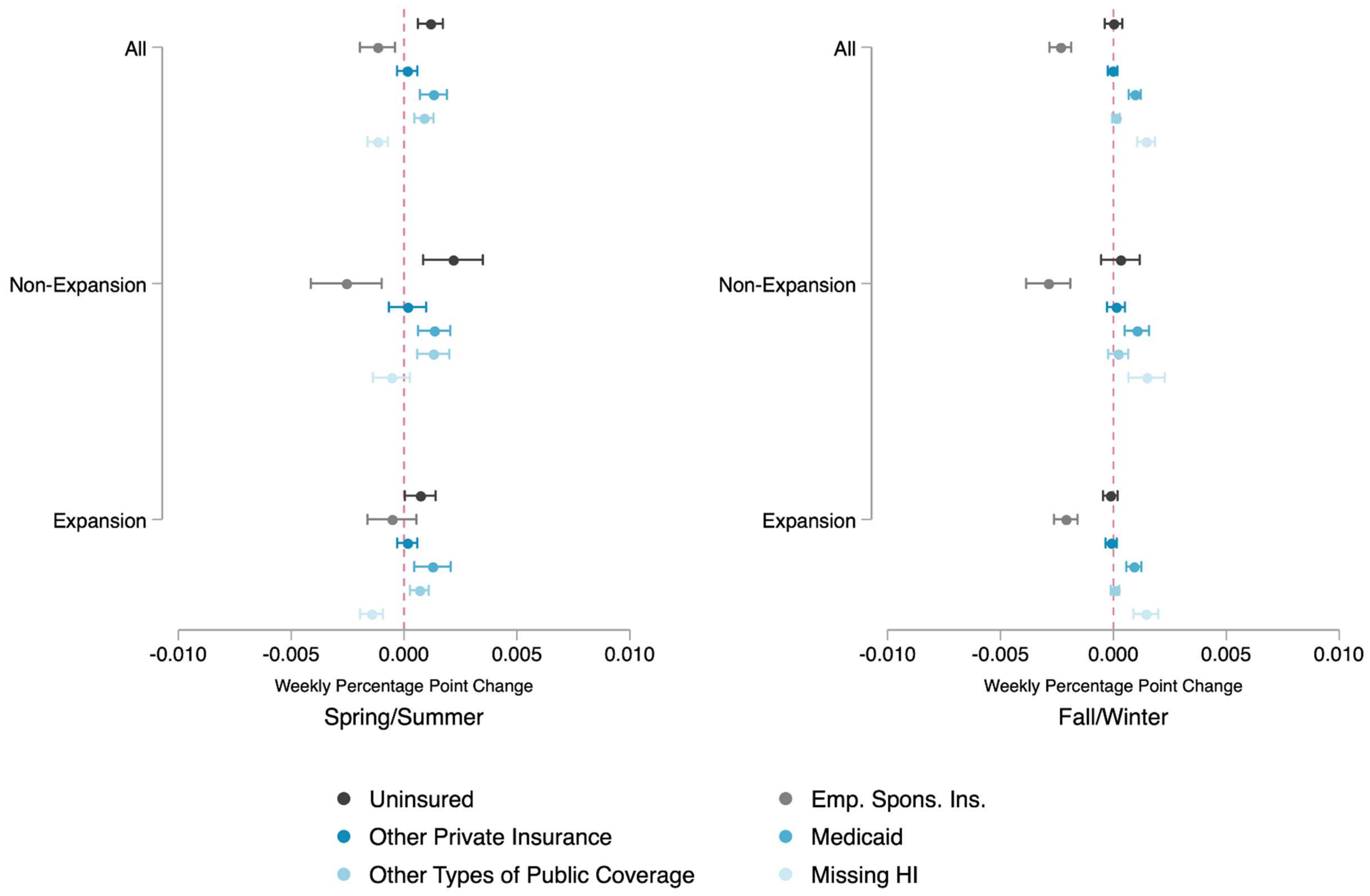

© 2021 Bundorf MK et al. *JAMA Health Forum*.

eFigure 4B: Sensitivity of Results to Dropping Missing Data, Demographic and Socioeconomic Characteristics

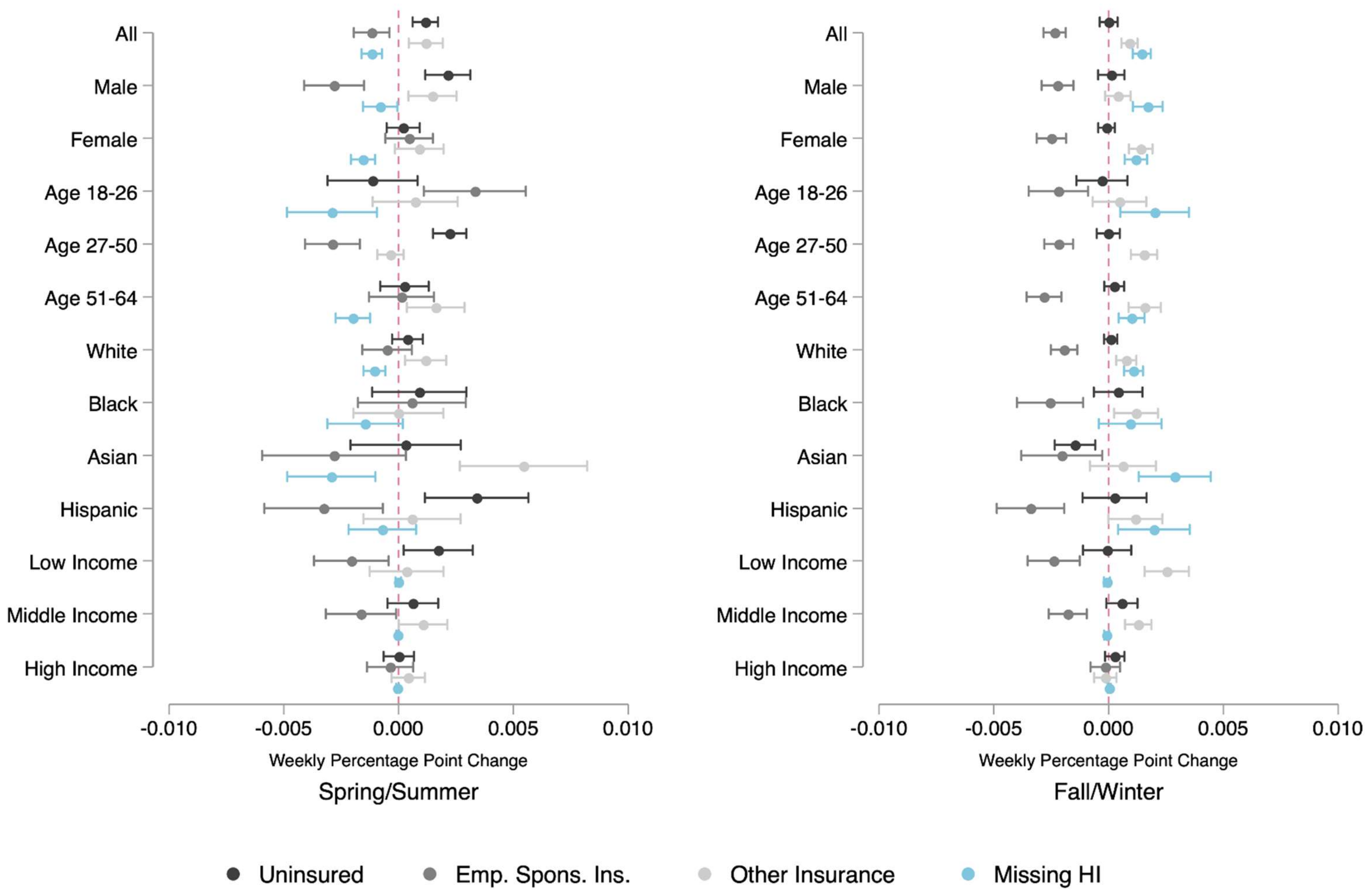

© 2021 Bundorf MK et al. *JAMA Health Forum*.

eTable 4A: Underlying Data for Estimates Presented in Figure 2

		Spring/Summer				Fall/Winter			
		Coverage Rate	Weekly Change	P-value	Standard Error	Coverage Rate	Weekly Change	P-value	Standard Error
All									
	Any Insurance	0.860	-0.0011	0.000	(0.0003)	0.865	-0.0003	0.170	(0.0002)
	ESI	0.654	-0.0021	0.000	(0.0004)	0.668	-0.0019	0.000	(0.0003)
	Non-ESI	0.206	0.0010	0.021	(0.0004)	0.197	0.0016	0.000	(0.0002)
	Other Private	0.070	0.0000	0.879	(0.0003)	0.071	0.0001	0.497	(0.0001)
	Medicaid	0.121	0.0013	0.000	(0.0003)	0.113	0.0015	0.000	(0.0002)
	Other Public	0.052	0.0009	0.000	(0.0002)	0.049	0.0002	0.031	(0.0001)
Non-Expansion States									
	Any Insurance	0.803	-0.0023	0.001	(0.0007)	0.810	-0.0007	0.163	(0.0005)
	ESI	0.622	-0.0032	0.000	(0.0008)	0.630	-0.0026	0.000	(0.0006)
	Non-ESI	0.181	0.0009	0.158	(0.0006)	0.180	0.0018	0.000	(0.0004)
	Other Private	0.079	0.0001	0.823	(0.0005)	0.081	0.0003	0.256	(0.0003)
	Medicaid	0.079	0.0014	0.001	(0.0004)	0.080	0.0015	0.000	(0.0003)
	Other Public	0.058	0.0014	0.001	(0.0004)	0.056	0.0004	0.191	(0.0003)
Expansion States									
	Any Insurance	0.885	-0.0006	0.116	(0.0004)	0.890	-0.0001	0.463	(0.0002)
	ESI	0.669	-0.0016	0.004	(0.0005)	0.685	-0.0016	0.000	(0.0003)
	Non-ESI	0.217	0.0010	0.063	(0.0005)	0.205	0.0015	0.000	(0.0003)
	Other Private	0.066	0.0000	0.912	(0.0003)	0.066	0.0000	0.970	(0.0002)
	Medicaid	0.140	0.0012	0.015	(0.0005)	0.128	0.0015	0.000	(0.0002)
	Other Public	0.049	0.0007	0.006	(0.0002)	0.046	0.0002	0.156	(0.0001)

Note: Authors' calculation of changes in health insurance coverage using the U.S. Census Household Pulse Survey (HPS). Estimates from linear regression of coverage on a continuous measure of calendar week, overall and in samples stratified by state Medicaid expansion status. The weekly change is the coefficient on the week variables. Standard errors, clustered by state, are in parentheses. Control variables include age, race and ethnicity, education, income, household size, the number of children in the household and state indicators. Estimate are weighted to be nationally representative using the replicate person-level weights provided by the HPS.

© 2021 Bundorf MK et al. *JAMA Health Forum*.

eTable 4B: Underlying Data for Estimates Presented in Figure 3

		Spring/Summer				Fall/Winter			
		Coverage Rate	Weekly Change	P-value	Standard Error	Coverage Rate	Weekly Change	P-value	Standard Error
Male	Any Insurance	0.841	-0.0023	0.000	(0.0005)	0.849	-0.0006	0.117	(0.0004)
	ESI	0.666	-0.0038	0.000	(0.0007)	0.679	-0.0015	0.000	(0.0004)
	Non-ESI	0.175	0.0014	0.020	(0.0006)	0.170	0.0010	0.005	(0.0003)
Female	Any Insurance	0.878	0.0000	0.919	(0.0004)	0.881	-0.0001	0.574	(0.0002)
	ESI	0.643	-0.0005	0.402	(0.0006)	0.658	-0.0023	0.000	(0.0004)
	Non-ESI	0.235	0.0005	0.359	(0.0006)	0.223	0.0022	0.000	(0.0003)
Age 18-26	Any Insurance	0.804	0.0021	0.058	(0.0011)	0.809	-0.0002	0.853	(0.0008)
	ESI	0.584	0.0019	0.122	(0.0012)	0.589	-0.0017	0.049	(0.0009)
	Non-ESI	0.219	0.0003	0.811	(0.0011)	0.221	0.0016	0.064	(0.0008)
Age 27-50	Any Insurance	0.847	-0.0025	0.000	(0.0004)	0.854	-0.0004	0.235	(0.0003)
	ESI	0.656	-0.0035	0.000	(0.0006)	0.670	-0.0016	0.000	(0.0003)
	Non-ESI	0.191	0.0010	0.076	(0.0006)	0.183	0.0013	0.000	(0.0003)
Age 51-64	Any Insurance	0.906	0.0000	0.964	(0.0006)	0.908	-0.0005	0.084	(0.0003)
	ESI	0.679	-0.0012	0.109	(0.0008)	0.697	-0.0027	0.000	(0.0004)
	Non-ESI	0.227	0.0012	0.077	(0.0007)	0.211	0.0022	0.000	(0.0004)
White	Any Insurance	0.896	-0.0003	0.456	(0.0004)	0.896	-0.0003	0.078	(0.0002)
	ESI	0.700	-0.0014	0.022	(0.0006)	0.707	-0.0016	0.000	(0.0003)
	Non-ESI	0.195	0.0011	0.031	(0.0005)	0.189	0.0013	0.000	(0.0003)
Black	Any Insurance	0.829	-0.0007	0.510	(0.0011)	0.835	-0.0009	0.202	(0.0007)
	ESI	0.587	-0.0002	0.894	(0.0014)	0.610	-0.0029	0.001	(0.0008)
	Non-ESI	0.243	-0.0005	0.639	(0.0011)	0.224	0.0020	0.002	(0.0006)
Asian	Any Insurance	0.902	-0.0001	0.942	(0.0013)	0.915	0.0015	0.010	(0.0006)
	ESI	0.720	-0.0055	0.002	(0.0017)	0.759	-0.0001	0.874	(0.0009)
	Non-ESI	0.182	0.0054	0.001	(0.0015)	0.155	0.0016	0.075	(0.0009)
Hispanic	Any private	0.754	-0.0039	0.002	(0.0012)	0.769	-0.0010	0.253	(0.0009)
	ESI	0.544	-0.0042	0.005	(0.0014)	0.560	-0.0032	0.000	(0.0008)
	Non-ESI	0.210	0.0002	0.851	(0.0012)	0.208	0.0022	0.007	(0.0008)
Low Income	Any Insurance	0.767	-0.0017	0.025	(0.0008)	0.771	0.0001	0.873	(0.0005)
	ESI	0.412	-0.0021	0.013	(0.0008)	0.419	-0.0024	0.000	(0.0006)
	Non-ESI	0.355	0.0003	0.672	(0.0008)	0.352	0.0025	0.000	(0.0005)
Middle Income	Any Insurance	0.889	-0.0006	0.270	(0.0006)	0.888	-0.0006	0.106	(0.0003)
	ESI	0.753	-0.0017	0.033	(0.0008)	0.748	-0.0018	0.000	(0.0004)
	Non-ESI	0.136	0.0011	0.051	(0.0005)	0.139	0.0013	0.000	(0.0003)
High Income	Any Insurance	0.963	0.0000	0.986	(0.0003)	0.960	-0.0003	0.208	(0.0002)
	ESI	0.891	-0.0004	0.411	(0.0005)	0.884	-0.0001	0.698	(0.0003)
	Non-ESI	0.072	0.0004	0.259	(0.0004)	0.076	-0.0001	0.558	(0.0002)

Note: Authors' calculation of changes in health insurance coverage during 2020 using the U.S. Census Household Pulse Survey (HPS). Estimates from linear regression of coverage on a continuous measure of calendar week, overall and in samples stratified demographic and socioeconomic characteristics. The weekly change is the coefficient on the week variables. Standard errors, clustered by state, are in parentheses. Control variables include age, race and ethnicity, education, income,

© 2021 Bundorf MK et al. *JAMA Health Forum*.

household size, the number of children in the household and state indicators. Estimate are weighted to be nationally representative using the replicate person-level weights provided by the HPS.

© 2021 Bundorf MK et al. *JAMA Health Forum*.

eReferences

[1] Health Insurance Coverage - Household Pulse Survey - COVID-19. Published June 30, 2021. Accessed July 2, 2021. https://www.cdc.gov/nchs/covid19/pulse/health-insurance-coverage.htm

[2] Cohen RA, Martinez ME. Health insurance coverage: Early release of estimates from the National Health Interview Survey, 2008: (565212009-001) [Internet]. American Psychological Association; 2009 [cited 2021 Jan 12]. Available from: http://doi.apa.org/get-pe-doi.cfm?doi=10.1037/e565212009-001

© 2021 Bundorf MK et al. *JAMA Health Forum*.
